# Comparison of access to health services among urban-to-urban and rural-to-urban older migrants, and urban and rural older permanent residents in Zhejiang Province, China: a cross-sectional survey

**DOI:** 10.1186/s12877-018-0866-4

**Published:** 2018-08-06

**Authors:** Sha Ma, Xudong Zhou, Minmin Jiang, Qiuju Li, Chao Gao, Weiming Cao, Lu Li

**Affiliations:** 1 The Institute of Social and Family Medicine, School of Medicine, Zhejiang University, 866 Yuhangtang Road, Xihu District, Hangzhou, Zhejiang Province 310058 People’s Republic of China; 2 School of Humanities and Social Sciences, Zhejiang Chinese Medical University, Gaoke Road, Fuyang District, Zhejiang Province 311402 People’s Republic of China

**Keywords:** Health needs, Health services, Health equity, Migrant older adults

## Abstract

**Background:**

While much literature reported the access of Chinese older migrants to health services, little was known about the differences among sub-groups of older adults, including urban-to-urban and rural-to-urban migrants, and urban and rural permanent residents. This study aimed to examine the access of these four groups to health services in Zhejiang Province, China and provide an evidence for the development of health services policies.

**Methods:**

A cross-sectional survey was conducted in community-dwelling older adults (aged 60 years or above) in 2013. Participants were recruited by random sampling. Demographic information and access to health services for the elderly populations were obtained via interviews using a self-designed structured questionnaire. Pearson’s chi-square tests and Cochran-Mantel-Haenszel (CMH) tests were performed to examine the differences in access to health services among the four groups. Binary logistic regression was conducted to explore the associations of participants’ visits to doctors with their group status after controlling confounding factors.

**Results:**

The two-week hospital visiting rates were significantly lower in migrants (55.56% in rural-to-urban and 62.50% in urban-to-urban) than that in urban and rural permanent residents (67.40 and 82.25%, respectively; *p* < 0.01). The majority of older adults who received a diagnosis indicating need for hospital treatment accepted the treatment, with no significant difference among the four groups after controlling for health service need (*χ*^*2*^ = 7.08, *p* = 0.07). On the other hand, 30.05% of the older adults did not visit a doctor when they got ailments in the past 2 weeks prior to the survey, and 16.42% (33/201) did not receive hospital treatment after receiving a diagnosis indicating need for hospital treatment. Factors including age, marital status, educational attainment, major financial source, and living with family members did not influence health services use.

**Conclusions:**

Targeted social and health policies integrating the strengths of government, society and families should be implemented to further improve health services use for different groups of older adults.

## Background

With continuing industrialization and urbanization, the number of older migrants in China has increased dramatically. Many of them move to cities to join their adult children and take care of their grandchildren. Immigration status is an important component in racial and ethnic disparities in access to healthcare [1]. Immigrant populations can easily become vulnerable groups in health services use in both developed and developing countries. A study in Europe reported that the migrants had less usage of screening and outpatient services for specialized care [2]. EU policy supports healthcare access for undocumented migrants, but practices remain haphazard [3]. A Czech study found that migrants were more likely to remain excluded from the public health insurance system [4]. Another study in Vietnam reported that the rural-to-urban older migrants had less access to health services [5]. Equity in health services means everyone has a chance to enjoy a fundamental preventive treatment, healthcare, and rehabilitation in need. Reported issues on health services accessibility included the following: i) The current status of health services use. A study conducted in Shanghai reported a low usage of health services in the older “floating” population [6]. ii) Accessibility to health services among vulnerable groups, including children [7], maternal women [8, 9], disabled people, migrants, people with mental illnesses [10], and prisoners [11]. iii) Barriers to health services, including previous negative experiences when accessing services [12], a long distance, a lack of knowledge regarding the location of government health facilities, and a lack of trust in government services [13]. iv) The influencing factors of access to health services on specific diseases, such as prediabetes [14], health insurance status, education, and gender [15].

To ensure health care for all people, China focused on provision of convenient and affordable health services across the rural and urban areas in previous decades and established a health insurance system consisting of health insurance for urban workers and residents, and the New Rural Cooperative Medical System (NCMS) for rural residents. Overall health insurance coverage was about 93% of its entire population in 2011, while urban residents had a greater access to health services via urban health insurance plans than those in the rural areas [16]. The majority of rural-to-urban migrants faced high public health risk due to their poor working and living conditions and lack of local social security and support [17]. A study containing a national sample reported that migrants had less access to public health services [18]. Another national survey showed that the weighted prevalence of healthcare service use was 36.6% in disabled elderly [19].

Previous Chinese studies focused on either migrant adults or older adults as a whole, but little was known about the differences in health services use among sub-groups including urban-to-urban and rural-to-urban older migrants, and urban and rural older permanent residents. Regarding health services use among these four groups in Zhejiang, we hypothesize that access to health and hospital services is universal. This study aimed to analyze access to health services and hospitalization in these four groups to explore whether older migrants are associated with lower access to health services after controlling for confounding factors. Besides, we want to identify the most vulnerable populations regarding health services use to provide a basis for promoting health policy development.

## Methods

### Sampling

Participants were divided into four groups of older adults: urban-to-urban migrants, rural-to-urban migrants, urban and rural permanent residents. Two-stage stratified cluster sampling was used to recruit urban older permanent residents from May to August 2013 in Hangzhou, Zhejiang Province, China. Gongshu, Jianggan, and Yuhang districts were randomly selected from 13 districts in Hangzhou to represent high, middle, and low levels of urbanization, respectively, and then one sub-district was randomly selected within each district. Two communities were randomly selected from each selected sub-district to represent a high or low economic level, resulting in six communities selected for the survey. A total of 1497 subjects, who lived in the six communities and met the study criteria, were surveyed and 1343 of them completed the questionnaires, of which 1322 were valid. The response rate was 89.71% and the validity rate was 98.44%.

Multi-stage stratified random sampling was conducted to select older migrants. Yuhang and Binjiang districts, which were migrant population centers, were selected from Hangzhou’s 13 districts. One sub-district was randomly selected from each of these two districts, and two communities were randomly selected from each of these sub-districts. A total of 1521 subjects were recruited, of which 1316 completed the questionnaire, with 1036 valid questionnaires (541 urban-to-urban and 495 rural-to-urban older migrants). The response rate was 86.52% and the validity rate was 78.72%.

Multi-stage stratified random sampling was adopted to extract the rural older permanent residents. Kaihua and Wuyi counties were selected as the sampling sites initially, and then one township was randomly selected from each county. Two administrative villages were randomly selected from each township, with 375 rural older permanent residents randomly selected from each village. A total of 1502 subjects were interviewed, 1468 completed the questionnaire, and 1449 questionnaires were valid, with the response rate of 97.74%, and the validity rate of 98.71%.

### Participants

Participants should met the following inclusion criteria: i) aged 60 years old and or above; ii) able to read, write, and communicate normally in Chinese; iii) without any mental illness; and iv) willing to participate in this study. Community or village leaders were asked to provide subjects who apparently met the requirements of the survey. Before the formal interview, investigators had an informal conversation with the subjects and excluded those subjects with a potential linguistic inability or mental illness. No differences were found among urban, rural, and migrant groups regarding their qualification to participate.

### Measures

A structured questionnaire was developed, covering socio-demographic characteristics, the need and use of health services. Data were collected via personal interviews and information was gathered anonymously. Socio-demographic characteristics included gender (female, male), age (60 to 69 years, 70 years or above), marital status (married, non-married), educational attainment (primary school or below, junior high school or above), main financial resource (oneself or their spouses, offspring and others), living with family members (yes, no), joined health insurance (yes, no), joined the endowment insurance (yes, no), and chronic diseases (yes, no).

In this study, health services accessibility was reflected by whether the subjects used health services when they had ailments or serious diseases. Firstly, we asked the participants whether they suffered from ailments in the past 2 weeks or an illness diagnosed by a doctor for hospitalization in the past year prior to the survey. These two items served as proxies for the need of health services. Health service need was measured by indicators including the two-week prevalence rate and the rate of diagnosis indicating need for hospital treatment in the past year. Indicators are calculated as below.

Two-week prevalence rate was equal to the number of participants who suffered from illness 2 weeks prior to the survey divided by the total number of participants surveyed within each group.

The diagnosis indicting need for hospital treatment rate in the past year was equal to the number of participants who should have received hospital treatment following a doctor’s diagnosis in the past year prior to the survey divided by the total number of participants surveyed within each group.

Then, we asked the participants with ailments whether they visited a doctor and asked those with a need for hospitalization whether they underwent to hospital treatment. These two items served as proxies for health service utilization. In another words, health service utilization was measured by indicators consisting of the two-week hospital visiting rate among participants with illness and the actual hospitalization rate among participants diagnosed for inpatient treatment. They are calculated as below.

The two-week hospital visiting rate among participants with illness was equal to the number of elderly who had visited a doctor in the past 2 weeks prior to the survey divided by the total number of the elderly with ailments within each group.

The hospitalization rate among the elderly diagnosed for inpatient treatment was equal to the number of elderly who had accepted hospital treatment in the past year prior to the survey divided by the total number of the elderly who should have received hospital treatment following doctor’s diagnosis within each group.

The reasons that the participants did not visit a doctor and go for hospital treatment in this study included mild symptoms, financial difficulty, lack of time, poor transportation system, unfamiliar with a place, self-treatment, lack of caregiver, and other reasons.

Urban older permanent residents refer to those aged 60 years and above with a registered permanent residence in Hangzhou who had lived in Hangzhou at least 6 months at the time of the survey. The older migrants were divided into urban-to-urban and rural-to-urban older migrants. The former refers to those aged 60 years or above who came from another city and lived in Hangzhou over 6 months at the time of the survey but did not have a registered permanent residence. The latter refers to people aged 60 years or above who came from a village and lived in Hangzhou over 6 months at the time of survey but did not have a registered permanent residence. The rural older permanent residents refer to those aged 60 years or above who had a rural registered permanent residence and had lived in that area for at least 6 months at the time of the survey.

### Statistical analysis

Statistical analysis was performed using the software SPSS 18.0. Descriptive statistics were used to describe the socio-demographic characteristics of the four groups of participants. Pearson’s chi-square tests and Cochran-Mantel-Haenszel tests were used to test the differences in access to health services among the four groups with health services need. Factors associated with participants’ visits to doctors were analyzed by binary logistic regression after controlling for socio-demographic characteristics. Statistical significance was set at *p* = 0.05 level.

## Results

### The socio-demographic characteristics of the participants

A total of 3807 participants were included in the analyses, consisting of 13.0% (495/3807) of rural-to-urban older migrants, 14.21% (541/3807) of urban-to-urban older migrants, 34.73% (1322/3807) of urban older permanent residents, and 38.06% (1449/3807) of rural older permanent residents. Their socio-demographic characteristics are shown in Table 1. The majority of participants aged 60 to 69 years, with differences between groups: urban-to-urban migrant group was 76.89%, rural-to-urban migrant group was 84.44%, and smaller portions of this age range were found in the urban (56.58%) and rural groups (51.97%). Proportion of the married participants was similar among groups (83.55% for the urban-to-urban migrant group, 80.20% for the rural-to-urban migrant group, 77.60% for the urban group, and 73.29% for the rural group). Those having an educational level of junior high school or above accounted for 46.32% in urban group, 31.31% in rural-to-urban group, and 9.39% in rural group, while it was 76.71% in the urban-to-urban group. Most participants depended on themselves or their spouses to live (85.58% in the urban-to-urban group, 77.99% in the urban group, 54.87% in the rural group, and 51.11% in the rural-to-urban group), and the others depended on their offspring or others. The majority in each of the four groups had health insurance, but this was significantly lower among the rural-to-urban group (79.80%) than the urban-to-urban group (90.57%), urban group (98.11%), and rural group (98.48%) (*χ*^*2*^ = 314.50, *p* < 0.01). More participants in the urban group (61.73%) had been diagnosed with a chronic disease than those in the other three groups (24.63% in urban-to-urban group, 26.63% in rural-to-urban group, and 49.55% in rural group).

**Table 1:** Socio-demographic and socio-economic characteristics of urban, urban-to-urban migrant, rural-to-urban migrant and rural older adults

Variables	Urban		Urban-to-urban		Rural-to-urban		Rural	
	n	%	n	%	n	%	n	%
Gender								
Male	530	40.09	271	50.09	208	42.02	708	48.86
Female	792	59.91	270	49.91	207	57.98	741	51.14
Age								
60~ 69	748	56.58	416	76.89	418	84.44	753	51.97
70 and above	574	43.42	125	23.11	77	15.56	696	48.03
Marriage status								
Marriage	1015	77.60	452	83.55	397	80.20	1062	73.29
Non-marriage	293	22.40	89	16.45	98	19.80	387	26.71
Educational attainment								
Primary school and below	707	53.68	126	23.29	340	68.69	1313	90.61
Junior high school and above	612	46.32	415	76.71	155	31.31	136	9.39
Major economic source								
Themselves or their spouse	1031	77.99	463	85.58	253	51.11	795	54.87
Offspring and others	291	22.01	78	14.42	242	48.89	654	45.13
Living with family members								
Yes	908	68.68	512	96.42	441	90.55	1097	75.71
No	414	31.32	19	3.58	46	9.45	352	24.29
Health insurance								
Participant	1297	98.11	490	90.57	395	79.80	1425	98.48
Nonparticipant	25	1.89	51	9.43	100	20.20	22	1.52
Chronic disease								
Yes	813	61.73	132	24.63	131	26.63	715	49.55
No	504	38.27	404	75.37	361	73.37	728	50.45

### Two-week prevalence rate

The difference in the two-week prevalence rate among the four groups was statistically significant (*χ*^*2*^ = 114.86, *p* < 0.01). The highest rate of physical discomfort in the past 2 weeks prior to the survey was reported by the urban group (24.81%; 326/1314) followed by the rural-to-urban group (13.56%; 67/494), while the rural and urban-to-urban migrant groups reported rates of 11.66% (169/1449) and 9.43% (51/541) respectively. Fewer males than females experienced ailments in urban group (odds ratio [OR] = 0.61, 95% confidence interval [CI] 0. 47 to 0.80) (Table 2). The subjects aged 60 to 69 had a higher rate than those aged 70 or above in urban group (OR = 2.30, 95% CI 1.70 to 3.12), while the differences between these two age categories among the other groups were not statistically significant. Married older adults had a lower ratio than the unmarried across four groups, and the difference was only statistically significant in the rural group (OR = 0.62, 95% CI 0.44 to 0.88). Participants with an education level of primary school or below had a lower risk than those with a higher education level in urban group (OR = 0.69, 95% CI 0.54 to 0.89). Subjects who depended on themselves or their spouses had a higher rate than those who depended on their offspring or others in the urban group (OR = 4.90, 95% CI 3.14 to 7.66). An opposite pattern was detected in the rural-to-urban and rural groups (OR = 0.56, 95%CI 0.33 to 0.95; OR = 0.64, 95%CI 0.46 to 0.88). For the urban subjects, those who lived with their family had a higher risk than those living alone (OR = 2.30, 95% CI 1.70 to 3.12). Subjects who were covered by health insurance had a lower risk than the others across four groups, with no statistical significance. Those with chronic disease had a higher risk than those without across four groups (OR = 11.17, 95% CI 7.29 to 17.12; OR = 14.98, 95% CI 7.39 to 30.36; OR = 12.43, 95% CI 6.73 to 22.99; OR = 7.03, 95% CI 4.53 to 10.90).

**Table 2:** Risk rate of two-week prevalence across demographic characteristics among urban, urban-to-urban, rural-to-urban, and rural old adults

Variables	Urban		Urban-to-urban		Rural-to-urban		Rural	
	OR	95%CI	OR	95%CI	OR	95%CI	OR	95%CI
Gender (Female)	0.61	0.47–0.80	1.13	0.64–2.02	1.51	0.90–2.54	0.96	0.70–1.32
Age (70 and above)	2.30	1.70–3.12	0.86	0.19–3.83	0.49	0.23–1.05	1.04	0.71–1.52
Marriage status (Non-marriage)	0.88	0.65–1.19	0.79	0.38–1.64	0.69	0.38–1.26	0.62	0.44–0.88
Educational attainment (Junior high school and above)	0.69	0.54–0.89	1.01	0.51–2.00	1.52	0.84–2.77	1.55	0.82–2.94
Major economic source (Offspring and others)	4.90	3.14–7.66	2.89	0.88–9.52	0.56	0.33–0.95	0.64	0.46–0.88
Living with family members (No)	2.30	1.70–3.12	0.86	0.19–3.83	0.49	0.23–1.05	1.04	0.71–1.52
Health insurance (No)	0.58	0.25–1.32	0.76	0.31–1.88	0.85	0.46–1.58	0.83	0.24–2.83
Chronic disease (No)	11.17	7.29–17.12	14.98	7.39–30.36	12.43	6.73–22.99	7.03	4.53–10.90

### Two-week hospital visiting rate among the elderly with illness

The difference in the two-week hospital visiting rate among the four groups of subjects with illness in the past 2 weeks was statistically significant (*χ*^*2*^ = 20.63, *p* < 0.01) after controlling for health service need. Older non-migrants were more likely to visit a doctor, the two-week hospital visiting rates for the rural group was 82.25% (139/169), 67.40% (215/319) for the urban group, 62.50% (30/48) for the urban-to-urban group and 55.56% (35/63) for the rural-to-urban group.

Males had a higher two-week hospital visiting rate than females among four groups, with no statistical significance (Table 3). Subjects aged 60 to 69 had a higher rate than subjects who aged 70 or above in the urban group (OR = 1.06, 95% CI 0.66 to 1.70), while the rate was lower for subjects aged 60 to 69 in urban-to-urban, rural-to-urban and rural groups, with no statistical significance. Married subjects in both urban and urban-to-urban groups had a higher rate than the unmarried, while married subjects in rural-to-urban and rural groups had a lower rate than the unmarried, with no statistical significance. Subjects with an education level of primary school or below had a lower rate than those with an education level of junior high school or above apart from the rural-to-urban group, with no statistical significance. Subjects who were economically independent or dependent on their spouses had a higher rate than the group who depended on their offspring or others in the rural-to-urban group; those who were economically independent or dependent on their spouses had a lower rate than the remaining, with no statistical significance. Except for the urban group, all subjects living with their family had a lower rate than those living alone, with no statistical significance. Those covered by health insurance had a lower rate than those without in rural group (OR = 0.82, 95% CI 0.76 to 0.88). Subjects with chronic disease had a lower rate than those without across four groups, with no statistical significance.

**Table 3:** Risk rate of two-week hospital visiting rate among the elderly with illness across demographic characteristics among urban, urban-to-urban, rural-to-urban, and rural old adults

Variables	Urban		Urban-to-urban		Rural-to-urban		Rural	
	OR	95%CI	OR	95%CI	OR	95%CI	OR	95%CI
Gender (Female)	1.64	1.00–2.69	1.00	0.31–3.22	1.26	0.46–3.42	1.30	0.59–2.87
Age (70 and above)	1.06	0.66–1.70	0.79	0.21–3.00	0.45	0.14–1.40	0.93	0.39–2.18
Marriage status (Non-marriage)	1.30	0.74–2.31	6.18	0.70–54.31	0.32	0.10–1.02	0.67	0.30–1.49
Educational attainment (Junior high school and above)	0.67	0.42–1.08	0.47	0.11–2.02	2.11	0.63–7.01	0.97	0.20–4.73
Major economic source (Offspring and others)	0.74	0.31–1.76	0.28	0.02–3.28	1.66	0.60–4.60	0.55	0.24–1.27
Living with family members (No)	1.12	0.61–2.04	0.59	0.46–0.76	0.51	0.13–2.01	0.67	0.28–1.61
Health insurance (No)	0.47	0.12–1.93	1.23	0.20–7.51	0.63	0.19–2.00	0.82	0.76–0.88
Chronic disease (No)	0.66	0.28–1.55	0.65	0.15–2.84	0.70	0.21–2.34	0.49	0.18–1.31

### Rate of diagnosis indicating need for hospital treatment in the past year

There was a statistically significant difference in the rate of diagnosis indicating need for hospital treatment among the four groups (*χ*^*2*^ = 88.5, *p* < 0.01). The rate was 11.16% (146/1308) for the urban group, 4.11% (22/535), 4.06% (20/493), and 3.03% (43/1420) for the urban-to-urban migrant, rural-to-urban migrant, and rural groups, respectively.

Male participants had a higher rate of diagnosis for hospitalization than the females in rural-to-urban and rural groups (OR = 4.39, 95% CI 1.57 to 12.28; OR = 2, 95% CI 1.06 to 3.78) (Table 4). Participants aged under 70 had a lower rate than those aged 70 or above in the rural group (OR = 0.48, 95% CI 0.25 to 0.90). Subjects with an educational level of primary school or below had a lower rate than those with an educational level of junior high school or above for urban and rural groups (OR = 0.61, 95% CI 0.43 to 0.86; OR = 0.43, 95% CI 0.19 to 0.95). Subjects who depended on themselves or their spouses had a higher rate than those who depended on their offspring or others both in urban group (OR = 5.55, 95% CI 2.69 to 11.47), while those who depend on themselves or their spouses had a lower rate than others in urban-to-urban (OR = 0.95, 95% CI 0.93 to 0.97). Subjects living with their family had a higher rate than those living alone both in the urban group (OR = 2.19, 95% CI 1.42 to 3.39). Subjects with chronic disease had a higher rate than those without among urban, urban-to-urban, rural-to-urban, and rural groups (OR = 5.45, 95% CI 3.24 to 9.16; OR = 11.95, 95% CI 4.31 to 33.09%; OR = 26.62, 95% CI 6.06 to 116.96; OR = 4.6, 95% CI 2.12 to 10.02).

**Table 4:** Risk rate of diagnosis indicating need for hospital treatment rate in the past year across demographic characteristics among urban, urban-to-urban, rural-to-urban, and rural old adults

Variables	Urban		Urban-to-urban		Rural-to-urban		Rural	
	OR	95%CI	OR	95%CI	OR	95%CI	OR	95%CI
Gender (Female)	1.08	0.76–1.53	1.78	0.74–4.33	4.39	1.57–12.28	2.00	1.06–3.78
Age (70 and above)	0.60	0.43–0.85	1.02	0.37–2.81	0.41	0.15–1.11	0.48	0.25–0.90
Marriage status (Non-marriage)	1.25	0.81–1.95	0.51	0.20–1.35	0.44	0.17–1.14	0.94	0.48–1.85
Educational attainment (Junior high school and above)	0.61	0.43–0.86	1.23	0.47–3.21	1.84	0.60–5.60	0.43	0.19–0.95
Major economic source (Offspring and others)	5.55	2.69–11.47	0.95	0.93–0.97	0.63	0.25–1.56	1.13	0.61–2.10
Living with family members (No)	2.19	1.42–3.39	0.74	0.09–5.83	2.04	0.27–15.57	0.67	0.35–1.28
Health insurance (No)	0.83	0.25–2.84	1.03	0.23–4.55	2.35	0.54–10.31	0.30	0.07–1.34
Chronic disease (No)	5.45	3.24–9.16	11.95	4.31–33.09	26.62	6.06–116.96	4.60	2.12–10.02

### The actual hospitalization rate among the elderly diagnosed for inpatient treatment

The majority of subjects who received a diagnosis indicating the need for hospital treatment accepted hospital treatment, with no statistically significant differences among the four groups after controlling for health service need (*χ*^*2*^ = 7.08, *p* = 0.07; the urban-to-urban group = 100% (19/19), rural-to-urban group = 95% (19/20), urban group = 80.74% (109/135), and rural group = 77.78% (21/27).

Male participants had a lower rate than females in urban group (OR = 0.34, 95% CI 0.13 to 0.92) (Table 5). Participants aged 60 to 69 had a lower rate than those aged 70 or above in the rural-to-urban and rural groups, while participants aged 60 to 69 in urban group had a higher rate than those aged 70 or above, with no statistical significance. Married participants had a lower rate than the unmarried in rural group (OR = 0.12, 95% CI 0.02 to 0.88). Participants with an educational level of primary school and below had a lower rate than those with junior high school education and above both in urban and rural groups, while participants with an educational level of primary school and below had a higher rate than those with an educational level of junior high school and above, with no statistical significance. Participants who depended on themselves or their spouses had a lower rate than those who depended on their offspring or others in urban group (OR = 0.80, 95% CI 0.73 to 0.87). Participants covered by health insurance had a lower rate than those without in rural group (OR = 0.77, 95% CI 0.62 to 0.95).

**Table 5:** Risk rate of the actual hospitalization rate among the elderly diagnosed for inpatient treatment across demographic characteristics among urban, urban-to-urban, rural-to-urban, and rural old adults

Variables	Urban		Urban-to-urban		Rural-to-urban		Rural	
	OR	95%CI	OR	95%CI	OR	95%CI	OR	95%CI
Gender (Female)	0.34	0.13–0.92	_	_	0.93	0.82–1.07	5.50	0.55–55.49
Age (70 and above)	1.09	0.46–2.57	_	_	0.93	0.80–1.07	0.27	0.03–2.70
Marriage status (Non-marriage)	0.93	0.31–2.77	_	_	0.92	0.79–1.08	0.12	0.02–0.88
Educational attainment (Junior high school and above)	0.52	0.21–1.31	_	_	1.33	0.76–2.35	0.21	0.02–1.97
Major economic source (Offspring and others)	0.80	0.73–0.87	_	_	0.88	0.67–1.14	0.31	0.05–2.08
Living with family members (No)	0.45	0.17–1.18	_	_	_	_	0.63	0.09–4.49
Health insurance (No)	0.47	0.04–5.36	_	_	0.94	0.84–1.06	0.77	0.62–0.95
Chronic disease (No)	1.77	0.38–8.31	_	_	0.94	0.84–1.06	0.94	0.08–10.87

### Barriers to use health services

Among 599 subjects with illness, 180 of them (30.05%) did not visit a doctor when they were uncomfortable (by group: 32.60% (104/319) urban, 37.50% (18/48) urban-to-urban migrant, 44.44% (28/63) rural-to-urban migrant, and 17.75% (30/169) rural). Subjects in each of the four groups faced different barriers to health services. Among the urban subjects, the main reasons for denial of health service were self-treatment (65.45%), other reasons (55.56%) and mild symptoms (52.94%). For the urban-to-urban subjects, the top three reasons for denial of health service were no time (25%), financial difficulty (16.67%), and mild symptoms (12.94%). For rural-to-urban subjects, the top three reasons were unfamiliar with the place (37.50%), other reasons (27.78%) and financial difficulty (16.67%). For the rural subjects, the top three reasons were scarce caregiver (66.67%), inadequate transportation (50%) and financial difficulty (38.89%).

16.42% (33/201) participants did not receive hospital treatment after receiving a diagnosis indicating the need for hospital treatment. The percentages of urban, urban-to-urban, rural-to-urban and rural older adults were 19.26% (26/135), 0% (0/19), 5% (1/20) and 22.22% (6/27) respectively. Across all three groups with participants who did not receive hospital treatment, the main reason given was mild symptoms (29.17% for urban subjects, 100% for rural-to-urban subjects and rural subjects).

### Factors influencing health services utilization

After controlling for socio-demographic characteristics including gender, age, marital status, educational attainment, major financial source, living with family members, health insurance, and having chronic diseases, rural subjects were more likely to visit doctors than urban subjects when they got illness in the past 2 weeks (OR = 2.29, *p* = 0.01) (Table 6).

**Table 6:** Binary logistic regression model to identify factors influencing two-week hospital visiting (including the person with ailment in the past two-week)

Variable (reference variable)	b	*p*	OR
Constant	2.10	0.02	8.18
Gender (female)	0.37	0.06	1.45
Age (70 and above)	−0.10	0.64	0.90
Marriage status (Non-marriage)	−0.08	0.74	0.92
Educational attainment (Junior high school and above)	−0.38	0.09	0.68
Major economic source (Offspring and others)	−0.15	0.60	0.86
Living with family members (No)	−0.14	0.58	0.87
Health insurance (No)	−0.29	0.49	0.75
Chronic disease (No)	−0.50	0.08	0.61
Category of older adults (Urban older adults)			
Urban-to-urban migrant older adults	−0.04	0.90	0.96
Rural-to-urban migrant older adults	−0.36	0.32	0.70
Rural older adults	0. 83	0.00	2.29

## Discussion

Many literature concerned with the access to health services for the migrant population [3, 20–22] tend to indicate that migrants have poorer access to health services [23, 24], while little difference had been found between migrants and non-migrants regarding health service use [25]. Little research had focused on older migrants, and the heterogeneity of older migrants was ignored in the one available publication comparing migrant with non-migrant population [5]. To our knowledge, the current study was the first to compare access to health services among four groups of older adults in China: urban-to-urban migrant, rural-to-urban migrant, urban, and rural older adults. Based on this special perspective, this study sheds a deeper understanding on the access to health services of four groups of elderly population and provides a more accurate basis for improving health welfare provision for older adults.

Previous research that indicated the low utilization of health services among migrant elderly [26–28], while this study found that the utilization of hospitalizations among older migrants were higher than non-migrant groups (not statistically significant). Compared with the hospitalization rate of 80.74% in urban elderly and 77.78% in rural elderly, the rates of both the urban-to-urban and rural-to-urban migrants were 100%. There are two reasons that can explain it.

First, in this study, urban-to-urban older migrants and rural-to-urban older migrants were younger and fewer of them had chronic disease when compared to the urban and rural groups. In the urban-to-urban group and the rural-to-urban group, the percentage of participants aged 60 to 69 were 76.89 to 84.44% respectively, while the percentages in the urban and rural groups were 56.58 and 51.97% respectively. Only 24.63% of urban-to-urban older adults and 26.63% rural-to-urban older adults had chronic diseases, while the percentage was 61.73% for the urban group and 49.55% in the rural group. Younger and healthier older adults were prone to accept hospital treatment in need. This opinion has been reported by a previous study, which found that younger and healthier older migrants would easily attach the importance to treatment when they were diagnosed with the need of hospitalization [2].

Second, a study found that high educational attainment can promote health service use for the older migrants [29]. In this study, urban-to-urban and rural-to-urban migrant groups had a higher educational attainment than urban and rural groups. 76.71% of urban-to-urban older migrants had an educational level of junior high school and above, which was higher than the urban group (46.32%). 31.31% of rural-to-urban migrants had an education level of junior high school and above, which was also higher than the rural group (9.39%). These could explain why older migrants were more likely to be hospitalized when they have been diagnosed as needing to be hospitalized.

Furthermore, this study found that the health insurance participation rate in rural group (98.48%) was the highest among four groups (98.11% urban, 90.57% urban-to-urban and 78.80% rural-to-urban groups). Meanwhile, rural elderly were more likely to visit a doctor when they got ailments (82.25%), but less of them were hospitalized (77.78%) when compared with other three groups. According to a previous study, rural elderly had a higher chance of visiting a doctor as they were benefited from China’s New-type Rural Cooperative Medical Scheme [30]. Over the past few decades, China has made important advances in achieving equal access to health services and insurance coverage across and within regions [31]. Meanwhile, China has basically achieved full coverage of health insurance [32].

Although four groups had high two-week prevalence rate and actual hospitalization rate, part of the older adults did not accept health service. Previous studies found that lack of community support [24], cultural beliefs and practices [33] are the major barriers for migrant elderly in accessing health services. However, this study found that the main reason why the older adults did not go for hospital treatment was because they did not identify the need for treatment. Four groups of older adults had different reasons for refusing a doctor’s visit when they got ailments. For the urban-to-urban and rural-to-urban migrant elderly, the main reasons were lack of time (25%) and being unfamiliar with the place (37.5%). While for the urban and rural groups, the main reasons for refusing a doctor’s visit were self-treatment and without caregiver. This suggested that a variety of social and health policies should be considered to improve the utilization of health services for different groups of older adults.

## Limitations

A limitation in this study was that no strict control group was established. The urban-to-urban and rural-to-urban older migrants came from different areas, which might have caused the heterogeneity in baseline health of the samples due to the economic development gaps between regions. Another limitation was that the cross-sectional study design can only allow for the collection of retrospective data; therefore, the postulation of the vulnerability of urban-to-urban and rural-to-urban older migrants in the near future needs to be further confirmed by prospective studies. Finally, we could not collect data on the intensity of disease, which weakened the assertions of the variations in health services utilization according to magnitude of need.

## Conclusions

Utilization of high health services among the four groups were detected in this study, while existing obstacles caused the low utilization of health services of older adults when they had ailments or serious diseases. According to the main obstacles of health service use in four groups, a variety of social and health policies integrating the strengths of government, society and family should be implemented for improving health service use in the future.

## Abbreviations

CI: Confidence interval
NCMS: the New Rural Cooperative Medical System
URBMI: the Urban Resident Basic Medical Insurance
